# The microbiome effect on the female reproductive performance

**DOI:** 10.1590/1984-3143-AR2024-0063

**Published:** 2024-08-12

**Authors:** Ricardo Zanella, Janine de Camargo, Claudia Almeida Scariot, Mariana Groke Marques

**Affiliations:** 1 Programa de Pós-graduação em BioExperimentação, Escola de Ciências Agrárias Inovação e Negócios, Universidade de Passo Fundo, Passo Fundo, RS, Brasil; 2 Embrapa Suínos e Aves, Concórdia, SC, Brasil

**Keywords:** vaginal microbial community, reproduction, fertility, 16S rDNA sequencing

## Abstract

The female reproductive function is coordinated by the endocrine system driven by the hypothalamic-pituitary-gonadal (HPG) axis. While not directly part of the female reproductive system, the gut microbiome plays a crucial role in overall health, including reproductive health. The gut microbiome communicates bidirectionally with the brain via the gut-brain axis, influencing stress levels, mood, and hormonal balance, which can impact reproductive health and fertility. In addition to that, the vaginal and uterine microbiome are directly involved with the reproductive success of farm animals, including female fertility and offspring development. In this paper, we summarize some of the effects of bacterial contamination in the female reproductive tract and their association with reproductive performance in farm animals.

## Introduction

The microbiome refers to the community of microorganisms that inhabit a particular environment, such as the animal body, soil, water, or even the atmosphere. These microorganisms include bacteria, fungi, viruses, archaea, protozoa, and other microscopic life forms (Berg et al., 2020; Facioli et al., 2020). In humans, it is estimated that over 57% of the genetic material in our bodies is nonhuman DNA, and microbial diversity constantly fluctuates between beneficial and harmful bacteria (Aggarwal et al., 2023). Microorganisms play crucial roles in various aspects of life, including animal health, environmental sustainability, and ecosystem functioning (Kaluanga Bwanga et al., 2023; Han et al., 2021; Inversetti et al., 2023).

For a long time, the uterus was characterized as a sterile reproductive organ (Kaluanga Bwanga et al., 2023). However, now it is known that the endometrial microbiome is composed of commensal bacteria, viruses, and yeast/fungi that appear to interact dynamically with the hormonal and immune regulation of the female reproductive tract (Han et al., 2021; Inversetti et al., 2023). The role of both the vaginal and gut microbiome has been studied in stress-related processes and their influence on the gut-brain axis and the hypothalamic-pituitary-adrenal (HPA) axis in the human (Rusch et al., 2023).

In humans, a healthy vaginal microbiota is typically dominated by *Lactobacillus* species including, *Lactobacillus crispatus, Lactobacillus gasseri, Lactobacillus iners,* and *Lactobacillus jensenii.* These vaginal lactobacilli, have been touted for their ability to prevent the invasion of pathogens by producing lactic acid and other compounds that help maintain an acidic pH environment (Chee et al., 2020). This acidic environment helps to inhibit the growth of harmful pathogens and maintain the balance of microorganisms in the vaginal ecosystem. When the balance of the vaginal microbiota is disrupted, it can lead to conditions such as bacterial vaginosis (BV), in which harmful bacteria outnumber beneficial bacteria like *Lactobacillus*. BV is associated with an increased risk of complications such as pelvic inflammatory disease, preterm birth, and other reproductive issues. In animals, maintaining a healthy vaginal microbiota is essential for reproductive success and overall health, as imbalances in the vaginal microbiota can lead to infertility, reproductive disorders, and increased susceptibility to infections (Kaambo et al., 2018).

Several factors, such as environment, physiology, genetics, and social relations, can modulate animal microbial diversity (Alves et al., 2022; Maraci et al., 2018). The human gut microbiome is now recognized as a key player in several phenotypic outcomes (ex., diseases, stress, reproduction, and mental health) (Ogunrinola et al., 2020). However, the mechanisms involved in the host and microbiota interaction in animals are still unclear.

## Microbial community in the sow reproductive tract

The vaginal microbiota plays a crucial role in maintaining the health of the female reproductive tract, including the uterus. The vaginal microbial diversity has significant implications for reproductive health, fertility, and pregnancy outcomes in sows (Poole et al., 2023). A study conducted by Sanglard et al. (2020) in gilts characterized a group of eighteen vaginal microbes and associated their different abundances with low and high farrowing rates. Sows with low reproductive performance had more harmful bacteria, such as *Phascolarctobacterium*, *Filifactor*, *Treponema*, and *Bacteroides,* than sows with high reproductive performance (Sanglard et al., 2020).

A recent work conducted by our group on sows has identified an effect of the housing condition on vaginal bacterial diversity and abundance. Interestingly, the animal welfare conditions were shown to be associated with greater vaginal microbial diversity, making the individuals more resilient to the disease (Alves et al., 2022), indicating that higher vaginal diversity correlates with healthier the uterine status. This is in contrast to what Sanglard et al. (2020) found, where they identified greater vaginal microbial diversity in the microbiome of sows with low reproductive performance compared to those with high reproductive performance. This could be explained by the different environmental and welfare conditions among the animals.

The characterization of vaginal microbiota and its interaction with reproductive success in animals is complicated since their composition can vary between individuals and can be influenced by factors such as animal genetics, age, hormonal status, diet, environmental factors, and housing conditions (Sanglard et al., 2020; Alves et al., 2022).

Another interesting finding is the potential impact of lysozyme supplementation in the diet of sows on their vaginal bacterial community. Lysozyme, an antimicrobial enzyme found naturally in the mucosal barrier of mammals, has been shown to influence the composition of vaginal microbiota (Xu et al., 2021). Specifically, the addition of lysozyme resulted in a decrease in the relative abundance of *Escherichia-Shigella* and an increase in *Lactobacillus* populations. *Lactobacillus* species protect against infections by other organisms and are considered potential probiotic candidates (Pino et al., 2021). This suggests that *Lactobacillus* dominance is associated with vaginal health and reproductive performance. The composition of the vaginal and cervix microbiota undergoes dramatic changes in response to the pregnantcondition of sows (Zhang et al., 2021). Further investigation by Zhang et al. (2021), found an effect of gut and vaginal microbiota on the estrus return of sows after weaning , identifying high counts if the *Bacteroidia, Lactobacillaceae*, and *Lactobacillus* in sows with normal return, while Actinobacteria, Clostridiales, Lachnospiraceae, Streptococcaceae, *Streptococcus*, *Clostridium*, *Mogibacterium*, *Ruminococcus*, and *Paludibacter* had higher abundances in non-return sows.

## Microbial community in the cow reproductive tract

In healthy cattle, the vaginal microbiota is typically composed of *Lactobacillus*, which like in humans, helps to inhibit the growth of harmful pathogens. Therefore, maintaining a healthy vaginal microbiota is particularly important in cattle breeding programs, as it can influence conception rates, embryo development, and pregnancy outcomes (Luecke et al., 2022). The imbalances in the vaginal microbiota can cause an overgrowth of pathogenic bacteria or a decrease in beneficial groups of microorganisms, which leading to reproductive disorders. The bacterial contamination of the animal's vaginal tract can have various effects depending on the type and abundance of bacteria involved, as well as the overall health status of the animal. It can disrupt the delicate balance of the vaginal microbiota, leading to conditions such as vaginitis, metritis, and endometritis. These inflammatory conditions can impair reproductive performance, including fertility, conception rates, and embryo survival (Quadros et al., 2020; Walsh et al., 2007).

The time-fixed artificial insemination protocol (TFAI) is a commonly used technique to synchronize estrus and ovulation in cattle. For that, the use of progesterone-releasing intravaginal device (PRID) in combination with other hormones is needed. While PRID is effective at manipulating reproductive processes in cattle, its use can potentially impact the vaginal microbiota resulting in vaginitis (Walsh et al., 2007). Quadros et al. (2020) suggested that the introduction of PRID directly affected the vaginal microbial composition in the Holstein cattle, creating a local environment and disrupting the natural microbial community in the vagina. Further investigation suggested the use of ceftiofur hydrochloride during the PRID removal to modulate the vaginal microbial changes.

Lof et al. (2018) demonstrated an improvement in pregnancy rates by 15% in beef cattle following the administration of 2.2 mg/kg of Ceftiofur via intramuscular route on the day of PRID removal. This indicates that the use of ceftiofur hydrochloride was effective in enhancing the reproductive efficiency of beef cattle undergoing TFAI. This indicates the critical role in the modulation of vaginal bacterial diversity and its importance in cattle reproductive success. Therefore, controlling the proliferation of vaginal bacteria or their introduction into the uterus during artificial insemination will help improve reproductive performance in cattle. Dias et al. (2019) showed the levels of vaginitis caused by the use of PRID impacted the pregnancy rates. The pregnancy rate associated with different scores of vaginitis was 60%, 57%, 52%, a nd 46% for scores 1, 2, 3, and 4, respectively. However, given the importance of that bacterial proliferation in maintaining homeostasis within the uterine and vaginal environment, it is possible that microbiota associated with reproductive tracts may significantly affect the reproductive success in cows (Mendez-Figueroa and Anderson, 2011). Women with bacterial vaginosis (BV) have a greater chance of infertility (van Oostrum et al., 2013). It is known that vaginitis can impact vaginal bacterial proliferation, and during artificial insemination, all these bacteria are carried to the uterus, possibly affecting sperm survivability and fertilization success (Liversedge et al., 1999). Recent data from our research group have shown a positive effect in the use of sanitary sheets during TFAI to prevent the introduction of vaginal contaminants to the uterus. An improvement of 16,7% in pregnancy rates in beef cattle and 17,6% in dairy cattle was identified in a group of animals inseminated using sanitary sheets (unpublished data). Furthermore, the uterine microbiota can indicate postpartum uterine health in cows, whereas cows with clinical endometritis had greater relative abundance of *Fusobacterium* and *Trueperella* lower relative abundance of *Escherichia Shigella, Lactobacillus, Prevotella, Schlegelella, Staphylococcus,* and *Streptococcus* than healthy cows. (Pascottini et al., 2020).

In other animal species, there is still no consensus on the exact microbiota composition of healthy females, as the environment has a greater impact on the microbial composition of livestock animals (Çömlekcioğlu et al., 2024).

Understanding the role of the microbiome in animals' reproductive performance is essential for implementing strategies to optimize reproductive efficiency and reduce the incidence of reproductive disorders. Management practices that promote a balanced microbiome, such as proper hygiene, dietary optimization, and antibiotics, can help support females' reproductive health and productivity. Additionally, further research is needed to elucidate the mechanisms by which the microbiome influences female reproductive performance and develop targeted interventions for improving fertility and litter outcomes.
